# Nomograms for predicting recurrence of HER2‐positive breast cancer with different HR status based on ultrasound and clinicopathological characteristics

**DOI:** 10.1002/cam4.70146

**Published:** 2024-09-09

**Authors:** Xudong Zhang, Hanqing Kong, Xiaoxue Liu, Qingxiang Li, Xinran Fang, Junjia Wang, Zihao Qin, Nana Hu, Jiawei Tian, Hao Cui, Lei Zhang

**Affiliations:** ^1^ Department of Abdominal Ultrasound the First Affiliated Hospital of Harbin Medical University Harbin Heilongjiang China; ^2^ Department of Ultrasound Medicine the Second Affiliated Hospital of Harbin Medical University Harbin Heilongjiang China; ^3^ Ultrasound molecular imaging Joint laboratory of Heilongjiang province (International Cooperation) Harbin Heilongjiang China

**Keywords:** HER2, breast cancer, ultrasound, nomogram, recurrence‐free survival

## Abstract

**Purpose:**

This study aimed to identify ultrasound and clinicopathological characteristics related to recurrence in HER2‐positive (HER2+) breast cancer, and to develop nomograms for predicting recurrence.

**Methods:**

In this dual‐center study, we retrospectively enrolled 570 patients with HER2+ breast cancer. The ultrasound and clinicopathological characteristics of hormone receptor (HR)−/HER2+ patients and HR+/HER2+ patients were analyzed separately according to HR status. Eighty percent of the original samples from HR−/HER2+ and HR+/HER2+ patients were extracted by bootstrap sampling as the training cohorts, while the remaining 20% were used as the external validation cohorts. Informative characteristics were screened through univariate and multivariable Cox regression in the training cohorts and used to develop nomograms for predicting recurrence. The predictive accuracy was calculated using Harrell's C‐index and calibration curves.

**Results:**

Three informative characteristics (axillary nodal status, calcification, and Adler degree) were identified in HR−/HER2+ patients, and another three (histological grade, axillary nodal status, and echogenic halo) in HR+/HER2+ patients. Based on these, two separate nomograms were constructed to assess recurrence risk. In the training cohorts, the C‐index was 0.740 (95% CI: 0.667–0.811) for HR−/HER2+ nomogram, and 0.749 (95% CI: 0.679–0.820) for HR+/HER2+ nomogram. In the validation cohorts, the C‐index was 0.708 (95% CI: 0.540–0.877) for HR−/HER2+ group, and 0.705 (95% CI: 0.557–0.853) for HR+/HER2+ group. The calibration curves also indicated the excellent accuracy of the nomograms.

**Conclusions:**

Ultrasound performance of HER2+ breast cancers with different HR status was significantly different. Nomograms integrating ultrasound and clinicopathological characteristics exhibited favorable performance and have the potential to serve as a reliable method for predicting recurrence in heterogeneous breast cancer.

## 1 INTRODUCTION

HER2‐positive (HER2+) breast cancer is characterized by a high degree of malignancy, prone to recurrence and metastasis.^1^ Previous studies have reported that 16%–22% of HER2+ breast cancer patients receiving adjuvant therapy will experience varying degrees of recurrence.^2^ It is evident that non‐invasive prediction of recurrence risk before surgery would contribute to guiding the formulation of treatment plans. Therefore, the assessment of recurrence risk in HER2+ breast cancer is extremely essential and valuable.

HER2+ breast cancer is a heterogeneous disease with various molecular biological expression.^3^ Notably, HER2+ patients with different hormone receptor (HR) status show significant differences in recurrence risk.^4^, ^5^ Therefore, in order to improve the accuracy of individualized prediction of recurrence in HER2+ breast cancer, it is necessary to analyze HR− patients and HR+ patients separately. Currently, intertumoral heterogeneity is reported to have a significant impact on diagnosis and prognosis, and is considered a potential prognostic factor.^6^, ^7^ However, it is difficult to evaluate differences in intertumoral heterogeneity by relying only on traditional clinical staging. Imaging characteristics can provide comprehensive tumor information and biological behavior. Therefore, images can offer a robust and non‐invasive method for characterizing intertumoral heterogeneity and assessing prognosis. To better understand the recurrence patterns of HER2+ breast cancer, additional biological and clinical markers are urgently needed to improve the accuracy of prediction in clinical practice.

In recent years, with the rapid development of imaging, many studies have assessed the prognosis of breast cancer by imaging characteristics. For instance, a radiomics‐based nomogram with texture characteristics from mammography has been developed to predict the prognosis in early‐stage TNBC.^8^ Additionally, some studies found that TNBC with vertical orientation in ultrasound was associated with poor prognosis.^9^ Furthermore, a few studies have predicted the recurrence of HER2+ breast cancer by volumetric‐tumor histogram‐based analysis of intravoxel incoherent motion and non‐Gaussian diffusion MRI.^10^ Ultrasound is the most common method for diagnosing and screening breast cancer, due to its advantages of convenience, non‐invasiveness, and non‐radiation. Therefore, assessing the recurrence of HER2+ breast cancer with different HR status through ultrasound images can provide more precise and personalized treatment, ultimately leading to significantly improved clinical outcomes.

As a statistical model, nomogram can predict the survival outcome of breast cancer based on multiple regression analysis, with the advantages being convenient, intuitive, and accurate. Nomogram can display weight scores for various clinical and radiological characteristics, and present the risk prediction values of multivariable parameters graphically.^11^ This approach has significant value for individualized diagnosis and treatment in clinical practice.

In summary, this study aims to identify ultrasound and clinicopathological characteristics related to the recurrence of HER2+ breast cancer, and construct HR−/HER2+ and HR+/HER2− nomograms, respectively, to assist with clinical prediction of disease recurrence.

## 2 METHODS

### 2.1 Patients

**FIGURE 1:**
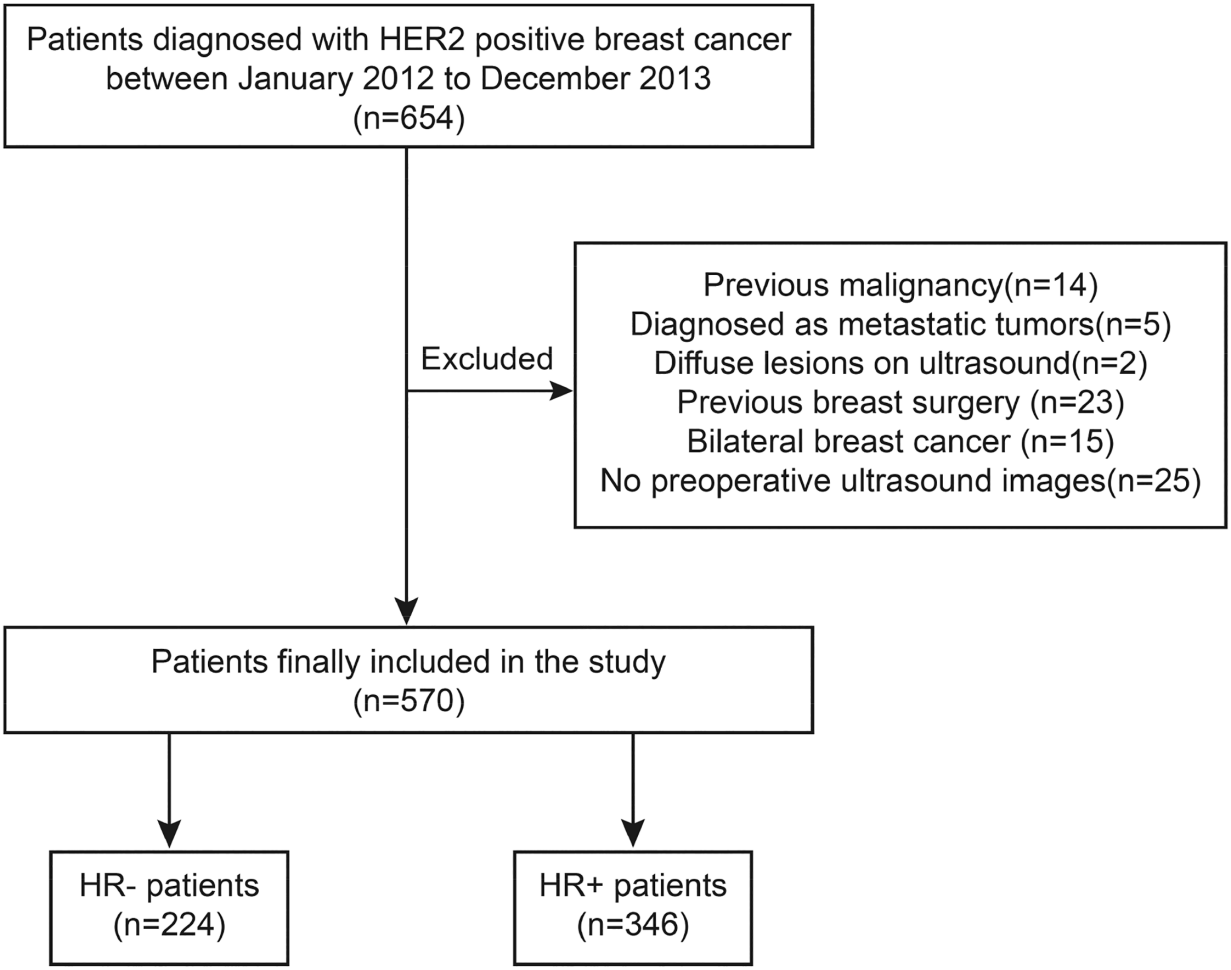
A flow chart of included HER2+ patients. A total of 570 patients were included in the study, among these, there were 224 HR− (ER− and PR−) patients and 346 HR+ (ER+ or PR+) patients.

Patients diagnosed with breast cancer between January 2012 and December 2013 who received treatment at the Second Affiliated Hospital of Harbin Medical University and Harbin Medical University Cancer Hospital were retrospectively enrolled in training and validation cohorts. This study was approved by the institutional ethics committee of Harbin medical university. The Ethics Committee waived the requirement for informed consent due to the retrospective study design. A total of 570 patients were included in the study. We separately analyzed HR−/HER2+ patients (*n* = 224) and HR+/HER2+ patients (*n* = 346) according to HR status. The flow chart for patient selection is presented in Figure 1.

### 2.2 Baseline data collection

Clinical information on age, menstrual history, lactation history, family history, and surgery type were collected through review of medical records. Pathological information on the tumor size, histological grade, axillary nodal status, and ER, PR, HER2, Ki‐67, P53, CK5/6, and E‐cadherin status were extracted from biopsy reports conducted before treatment. For immunohistochemistry (IHC), an ER+ or PR+ label required at least 1% positive nuclear staining in the tumor.^12^ A HER2+ designation required a HER2 score of 3+ or 2+, and positive fluorescence in situ hybridization; a HER2 score of 0 or 1+ was defined as HER2−.^13^ The cutoff value for the high expression of Ki‐67 was 14%.^14^

### 2.3 Ultrasound examination

The ultrasound images of breast lesions were detected and stored using a HI VISION AVIUS system (Hitachi, Japan) equipped with a 5–12 MHz linear probe for retrospective analysis. All ultrasound images were not otherwise pre‐processed except to remove patient information. Two ultrasound experts, each with over 5 years of experience in breast ultrasound, analyzed the ultrasound characteristics in a double‐blind manner. In case of disagreement, the two experts discussed together and reached a consensus. The ultrasound characteristics were extracted according to ACR BI‐RADS Atlas (2013) (Table S1).^15^ In addition, the Adler degree of breast lesions was assessed.^16^

### 2.4 Patients follow‐up and definition of censored patients

Patients' follow‐up protocol: All patients were followed up through outpatient reviews or telephone contacts every 1–3 months in the first 2 years continuously, and then every 6 months thereafter. Locoregional recurrence or distant metastasis was diagnosed through imaging and pathological examinations.

Definition of censored patients: we selected recurrence‐free survival (RFS) as the endpoint.^12^, ^17^, ^18^ RFS was calculated from the initial treatment date until the occurrence of disease progression (locoregional recurrence or distant metastasis), death or the most recent follow‐up date.

### 2.5 Development of nomogram model

**FIGURE 2:**
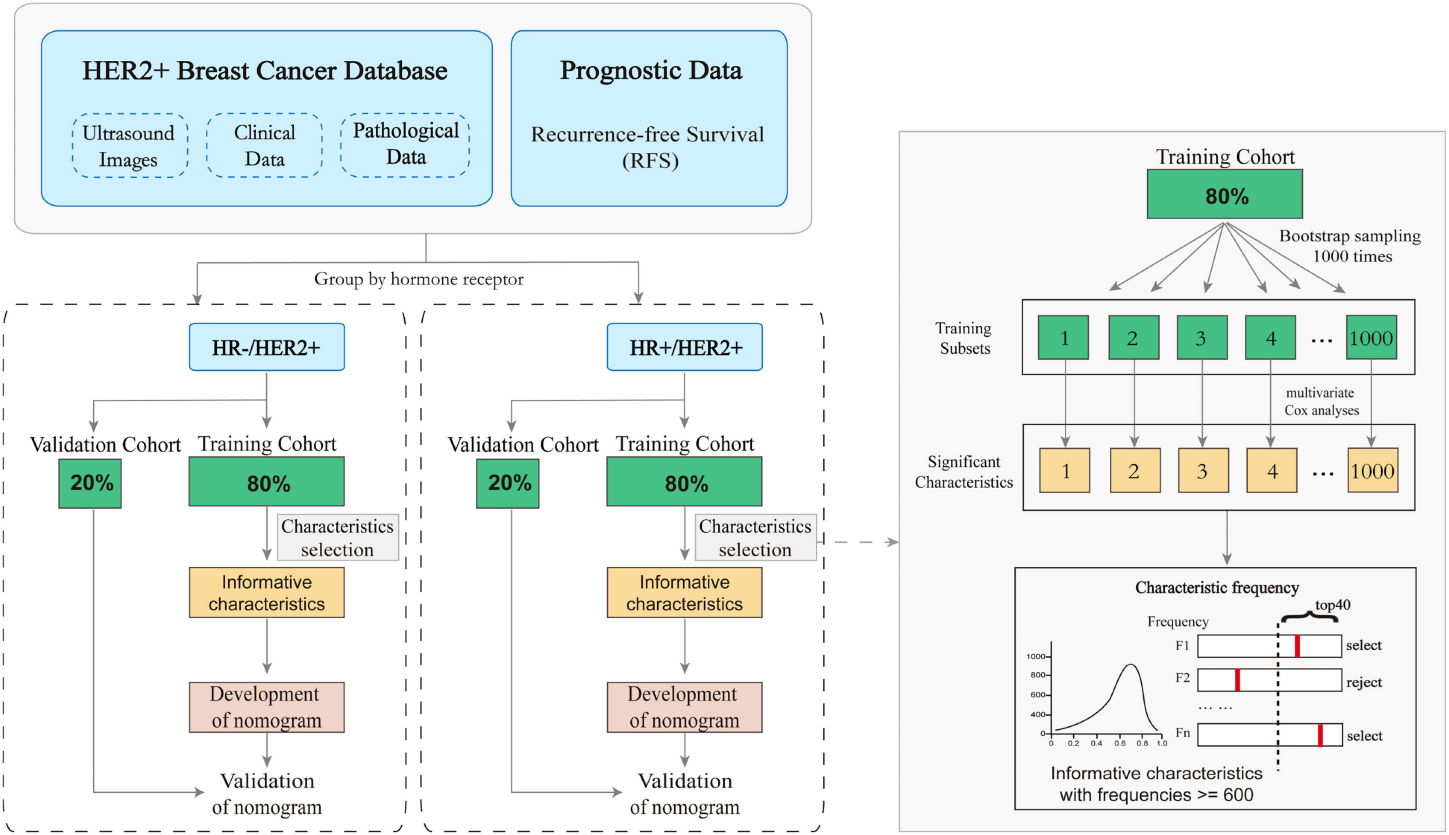
Workflow of extracting informative characteristics from ultrasound images and clinicopathological information to develop two nomograms.

We randomly selected 80% of the original samples from HR−/HER2+ and HR+/HER2+ patients as the training cohort, and the remaining 20% as the external validation cohort, respectively. Subsequently, 1000 training subsets were generated from the training cohort through bootstrap sampling (1000 times). The significant characteristics related to recurrence were screened by univariate Cox regression analysis in each training subset. The characteristics with *p* < 0.2 were further analyzed by multivariable analysis. The significant characteristics with a frequency greater than 600 in the 1000 training subsets were regarded as informative characteristics. Based on the above, two nomograms were developed to assess the recurrence of HR−/HER2 and HR+/HER2 breast cancer, respectively. Details of the work flow for building the model are shown in Figure 2.

Then, the Kaplan–Meier plots of informative characteristics for HR−/HER2+ and HR+/HER2+ groups were developed. The informative characteristics were weighted according to their respective coefficients and linearly combined to construct the rad‐score calculation formula.^19^ Patients were divided into high‐risk and low‐risk groups based on median rad‐score, and the two groups were compared for recurrence.

### 2.6 Validation of nomogram model

The Harrell's C‐index was used to evaluate the performance of nomograms, and the calibration curve was drawn to evaluate the calibration effect.^20^ The value of the C‐index can range from 0.5 (indicating indiscriminate ability) to 1.0 (indicating a complete distinction between patients with disease progression or death and those without disease progression or death).

### 2.7 Statistical analysis

R Version 4.1.1 (http://www.R‐project.org) was used for statistical analysis. R packages included are as follows: the “survival” package was used for Cox proportional hazards regression; the “survival” and “ggplot2” package was used for Kaplan–Meier survival analyses; the “rms” package was used for nomograms and calibration curves. Pearson's Chi‐square test and independent *t*‐test were used for comparison between the training cohort and validation cohort. All statistical tests were two‐sided, and *p* < 0.05 was considered statistically significant.

## 3 RESULTS

### 3.1 Baseline data

A total of 570 HER2+ patients were included in the study, comprising 224 HR− patients and 346 HR+ patients. In terms of clinicopathology, 10 characteristics showed significant differences (*p* < 0.021) between HR−/HER2+ and HR+/HER2+ groups, except for four (*p* = 0.074–0.989), as presented in Table 1. In terms of ultrasound, there were significant differences in eight characteristics (*p* < 0.004), except for orientation and internal echo (*p* = 0.581 and *p* = 0.105, respectively) (Table 2). The comparison between the training cohort and the validation cohort is shown in Tables S2 and S3.

**TABLE 1:** Clinicopathological characteristics of patients.

Characteristic	Total (*n* = 570)	HR−/HER2+ (*n* = 224)	HR+/HER2+ (*n* = 346)	*p*‐value
Age (years)				
≤35	338 (59.3)	161 (71.9)	177 (51.2)	<0.001
>35	232 (40.7)	63 (28.1)	169 (48.8)	
Menstrual history				
Normal	401 (70.4)	204 (91.1)	197 (56.9)	<0.001
Abnormal	169 (29.6)	20 (8.9)	149 (43.1)	
Family history				
Absent	389 (68.2)	124 (55.4)	265 (76.6)	<0.001
Present	181 (31.8)	100 (44.6)	81 (23.4)	
Surgery type				
BCT	28 (4.9)	6 (2.7)	22 (6.4)	0.074
Mast	542 (95.1)	218 (97.3)	324 (93.6)	
Lactation history				
Absent	236 (41.4)	130 (58)	106 (30.6)	<0.001
Present	334 (58.6)	94 (42)	240 (69.4)	
Tumor size				
≤2 cm	346 (60.7)	122 (54.5)	224 (64.7)	0.018
>2 cm	224 (39.3)	102 (45.5)	122 (35.3)	
Histological grade				
1–2	386 (67.7)	108 (48.2)	278 (80.3)	<0.001
3	184 (32.3)	116 (51.8)	68 (19.7)	
Axillary nodal status				
No involvement	252 (44.2)	133 (59.4)	119 (34.4)	<0.001
Involvement	318 (55.8)	91 (40.6)	227 (65.6)	
Ki67				
≤30	235 (41.2)	112 (50)	123 (35.5)	<0.001
>30	335 (58.8)	112 (50)	223 (64.5)	
P53				
Negative	214 (37.5)	122 (54.5)	92 (26.6)	<0.001
Positive	356 (62.5)	102 (45.5)	254 (73.4)	
CK5/6				
Negative	310 (54.4)	126 (56.2)	184 (53.2)	0.527
Positive	260 (45.6)	98 (43.8)	162 (46.8)	
E‐cadherin				
Negative	345 (60.5)	135 (60.3)	210 (60.7)	0.989
Positive	225 (39.5)	89 (39.7)	136 (39.3)	
CEA				
Negative	312 (54.7)	125 (55.8)	187 (54)	0.745
Positive	258 (45.3)	99 (44.2)	159 (46)	
CA153				
Negative	262 (46)	89 (39.7)	173 (50)	0.021
Positive	308 (54)	135 (60.3)	173 (50)	

Abbreviations: BCT, breast conservation therapy; Mast, mastectomy.

**TABLE 2:** Ultrasound characteristics of the breast lesions.

Characteristic	Total (*n* = 570)	HR−/HER2+ (*n* = 224)	HR+/HER2+ (*n* = 346)	*p*‐value
Shape				
Regular	209 (36.7)	116 (51.8)	93 (26.9)	<0.001
Irregular	361 (63.3)	108 (48.2)	253 (73.1)	
Orientation				
Parallel	301 (52.8)	122 (54.5)	179 (51.7)	0.581
Vertical	269 (47.2)	102 (45.5)	167 (48.3)	
Boundary				
Circumscribed	101 (17.7)	26 (11.6)	75 (21.7)	0.003
Indistinct	469 (82.3)	198 (88.4)	271 (78.3)	
Margin				
Smooth	326 (57.2)	111 (49.6)	215 (62.1)	0.004
Lobulate	244 (42.8)	113 (50.4)	131 (37.9)	
Posterior acoustic pattern				
No enhancement	270 (47.4)	156 (69.6)	114 (32.9)	<0.001
Enhancement	300 (52.6)	68 (30.4)	232 (67.1)	
Calcification				
Absent	248 (43.5)	147 (65.6)	101 (29.2)	<0.001
Present	322 (56.5)	77 (34.4)	245 (70.8)	
Echogenic halo				
Absent	128 (22.5)	25 (11.2)	103 (29.8)	<0.001
Present	442 (77.5)	199 (88.8)	243 (70.2)	
Internal echo				
Hypoechoic	267 (46.8)	95 (42.4)	172 (49.7)	0.105
Mixed‐echoic	303 (53.2)	129 (57.6)	174 (50.3)	
Adler degree				
0–1	340 (59.6)	80 (35.7)	260 (75.1)	<0.001
2–3	230 (40.4)	144 (64.3)	86 (24.9)	
BI‐RADS				
≤3	383 (67.2)	195 (87.1)	188 (54.3)	<0.001
>3	187 (32.8)	29 (12.9)	158 (45.7)	

### 3.2 Recurrence status of HR−/HER2+ and HR+/HER2+ patients

In this study, all patients were followed up from 6 to 60 months (median, 36 months). Among the 570 patients, 95 cases (16.7%) experienced disease recurrence, while the remaining 475 cases (83.3%) did not experience recurrence. Of the 95 patients with recurrence, 44 cases were HR−/HER2+, and 51 cases were HR+/HER2+ (Table 3).

**TABLE 3:** Survival data of HR−/HER2+ and HR+/HER2+ patients.

	Total (*n* = 570)	HR−/HER2+ (*n* = 224)	HR+/HER2+ (*n* = 346)	*p*‐value
Status				
No recurrence	475 (83.3)	180 (80.4)	295 (85.3)	0.156
Recurrence	95 (16.7)	44 (19.6)	51 (14.7)	
RFS (month)				
Median	36 (6–60)	35 (6–60)	37 (6–60)	0.814

Abbreviation: RFS, recurrence‐free survival.

### 3.3 Informative characteristics associated with recurrence in HR−/HER2+ and HR+/HER2+ patients

**FIGURE 3:**
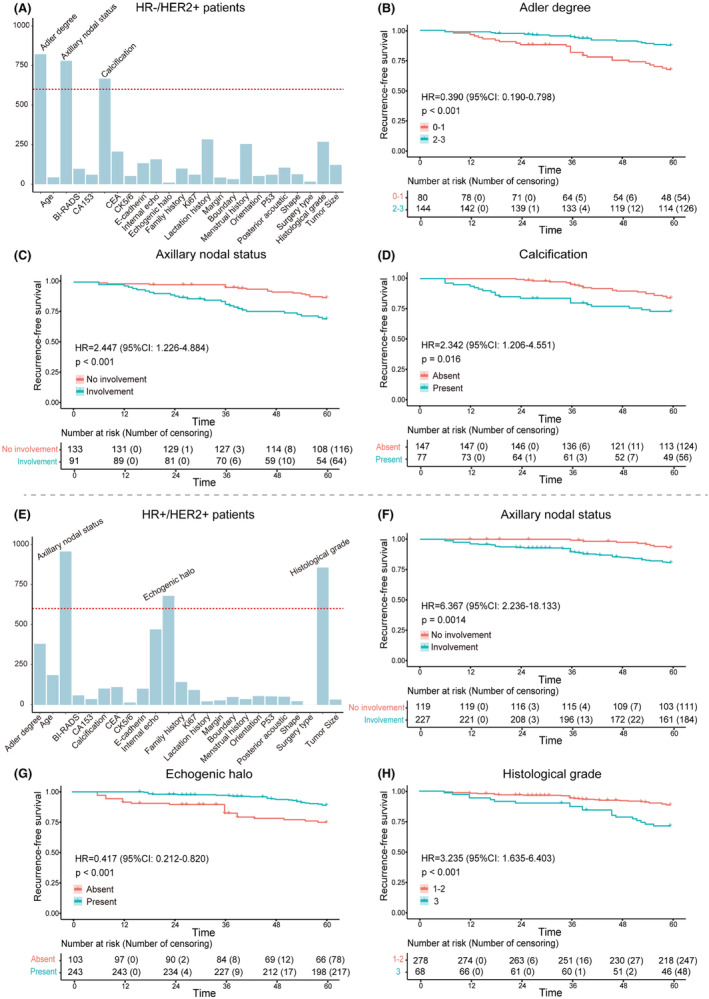
(A–D) The informative characteristics with a frequency greater than 600 in the 1000 training subsets of HR−/HER2+ group, and their corresponding Kaplan–Meier plots for RFS. (B–D) Axillary nodal status, calcification, and Adler degree were strongly associated with RFS in HR−/HER2+ patients. (E–H) The informative characteristics with a frequency greater than 600 in the 1000 training subsets of HR+/HER2+ group, and their corresponding Kaplan–Meier plots for RFS. (F–H) Histological grade, axillary nodal status, and echogenic halo were highly associated with RFS in HR+/HER2+ patients.

Univariate and multivariable Cox regression analyses were performed in the training cohorts of HR−/HER2+ and HR+/HER2+ patients, respectively (Tables S4 and S5). The distribution of each characteristic in the 1000 training cohorts of HR−/HER2+ and HR+/HER2+ is shown in Figure 3. For HR−/HER2+ patients, there were three characteristics with frequency greater than 600 times, including axillary nodal status (759 times), calcification (651 times), and Adler degree (914 times) (Figure 3A). In addition, three informative characteristics were also screened out in HR+/HER2+ patients, including histological grade (911 times), axillary nodal status (966 times), and echogenic halo (881 times) (Figure 3E).

**FIGURE 4:**
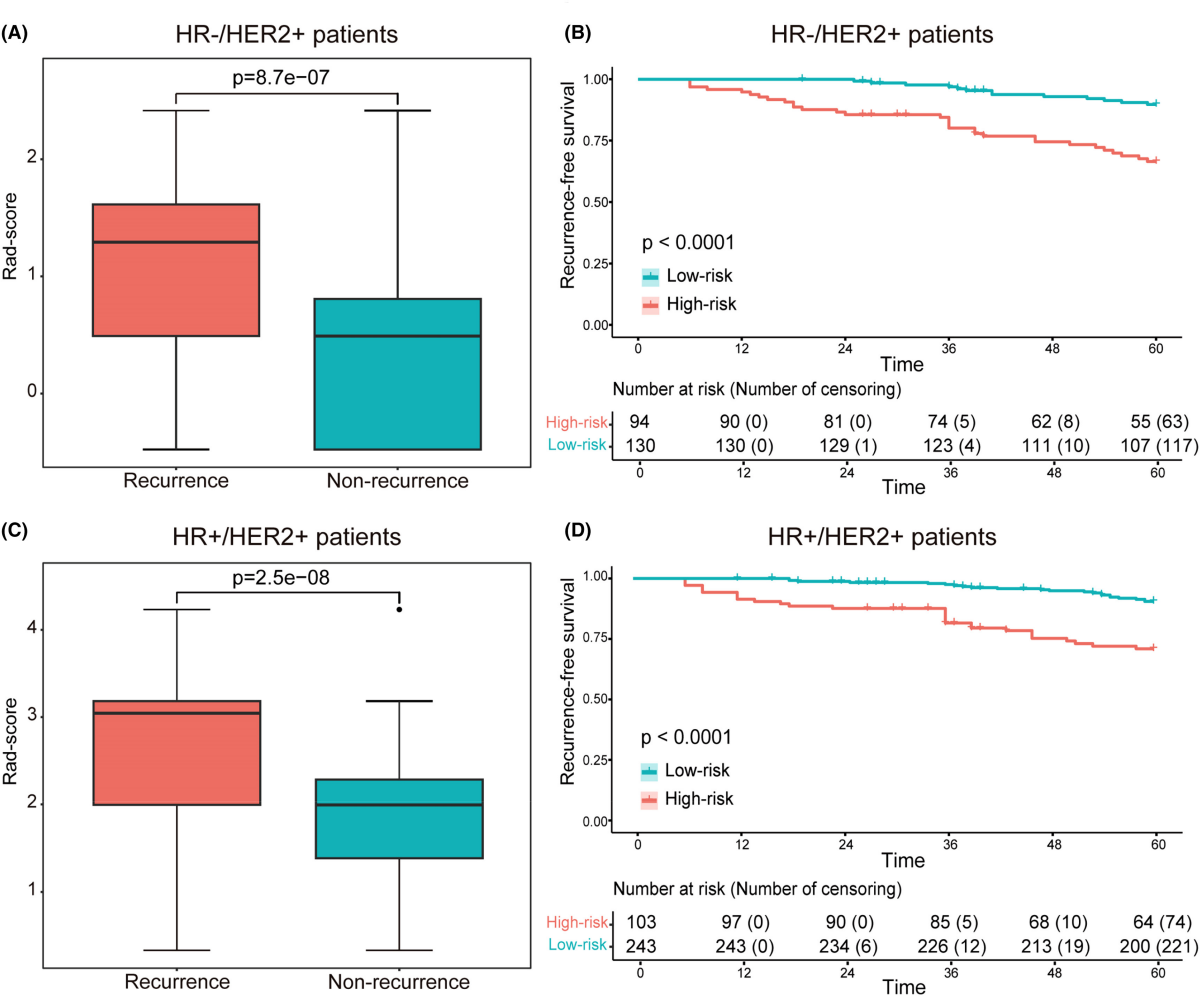
(A, C) The box plots comparing rad‐score between the recurrence and non‐recurrence groups in HR−/HER2+ and HR+/HER2+ patients, respectively. (B, D) The survival curves of RFS with low‐risk and high‐risk were evaluated in the HR−/HER2+ and HR+/HER2+ groups, respectively.

Kaplan–Meier plots demonstrated excellent results for each informative characteristic selected in both HR−/HER2+ patients and HR+/HER2+ patients, with *p* values well below 0.05 (Figure 3B–D,F–H). Additionally, the rad‐score of each patient was calculated based on the coefficients of informative characteristics obtained through multivariable Cox analysis in the training cohorts. Figure 4A,C, respectively show the differences in rad‐score between the recurrence and non‐recurrence groups, indicating higher rad‐score among recurrent patients. Furthermore, patients were divided into high‐risk group and low‐risk group based on the median rad‐score of the training cohort. Kaplan–Meier plots showed there were significant differences between high‐risk and low‐risk patients (Figure 4B,D).

### 3.4 The nomograms for predicting RFS

**FIGURE 5:**
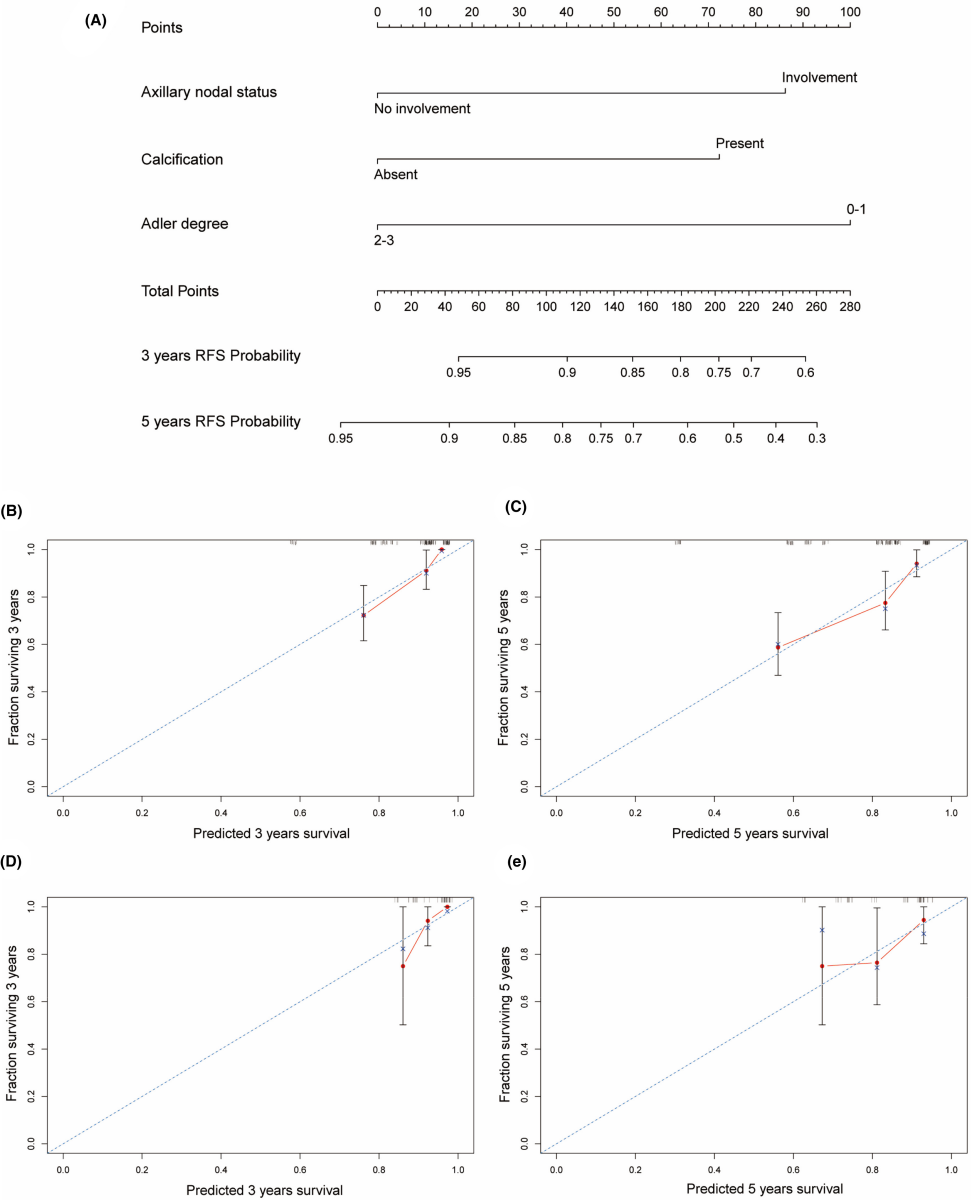
Nomogram for HR−/HER2+ patients RFS with calibration curves. (A) Nomogram of HR−/HER2+ for 3 and 5‐years; (B, C) Calibration curves for 3 and 5‐years in the training cohorts; (D, E) Calibration curves for 3 and 5‐years in the validation cohorts.

**FIGURE 6:**
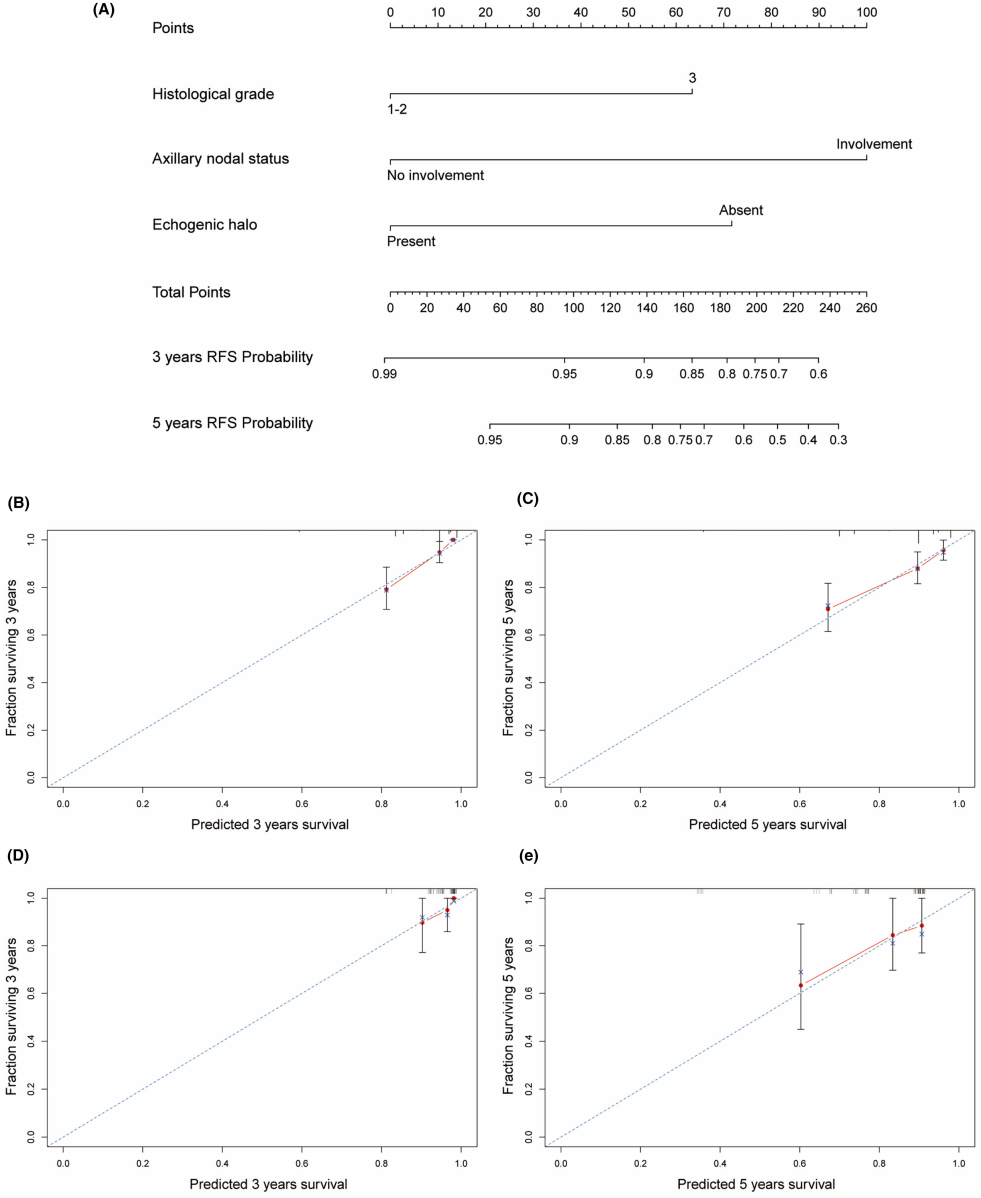
Nomogram for HR+/HER2+ patients RFS with calibration curves. (A) Nomogram of HR+/HER2+ for 3 and 5‐years; (B, C) Calibration curves for 3 and 5‐years in the training cohorts; (D, E) Calibration curves for 3 and 5‐years in the validation cohorts.

Based on the informative characteristics of HR−/HER2+ and HR+/HER2+ patients, we developed two nomograms to assess the risk of recurrence, respectively. In HR−/HER2+ nomogram, the probability of recurrence will be higher in patients with axillary lymph node involvement, ultrasound images showing calcification, and lower Adler degree (Figure 5). In HR+/HER2+ group, the risk of recurrence will be higher in patients with axillary lymph node involvement, higher histological grade, and no echogenic halo on ultrasound images (Figure 6). Furthermore, the predictive accuracy of the models was evaluated through the C‐index. In the training cohort, the C‐index of the nomogram was 0.740 (95% CI: 0.667–0.811) for HR−/HER2+ group, and 0.749 (95% CI: 0.679–0.820) for HR+/HER2+ group. In the validation cohort, the C‐index for the nomogram was 0.708 (95% CI: 0.540–0.877) for HR−/HER2+ group, and 0.705 (95% CI: 0.557–0.853) for HR+/HER2+ group. Additionally, the high‐quality calibration curves in both HR−/HER2+ and HR+/HER2+ nomograms were evaluated.

## 4 DISCUSSION

In this study, clinicopathological and ultrasound characteristics associated with recurrence of HER2+ breast cancer were screened. Based on these characteristics, two nomograms were constructed to assess the recurrence of HR−/HER2 and HR+/HER2 breast cancer, respectively. Previous studies have demonstrated the differences in disease progression between HR−/HER2+ and HR+/HER2+ patients.^6^, ^7^ Therefore, HER2+ breast cancer was divided into two groups according to HR status for our research. We found that the ultrasound and clinicopathological characteristics were significantly different between HR−/HER2+ and HR+/HER2+ patients. In HR−/HER2+ group, patients with calcification and lower Adler degree on ultrasound, and axillary lymph node metastasis were more prone to recurrence. In HR+/HER2+ group, patients with the absence of echogenic halo on ultrasound, axillary lymph node metastasis, and high histological grade were more likely to relapse. Moreover, the nomograms with excellent prediction performance were constructed based on these informative characteristics.

In terms of clinicopathology, we concluded that axillary lymph node metastasis is closely related to the recurrence of HER2+ breast cancer. Axillary lymph node status, as an independent prognostic factor, is critical for breast cancer prognosis.^21^ Axillary lymph node metastasis serves as the primary route of breast cancer spread.^22^ Therefore, in HER2+ breast cancer, regardless of HR status, axillary lymph node metastasis is an important biomarker for poor prognosis. We also found that patients with high histological grade often experienced recurrence in HR+/HER2+ group. As another important prognostic factor for breast cancer, histological grade represents a morphological assessment of tumor biological characteristics and can accurately predict tumor biological behavior.^23^ Studies have demonstrated that histological grade is correlated with RFS in ER+ breast cancer and can provide important prognostic information for clinical treatment.^24^, ^25^

**FIGURE 7:**
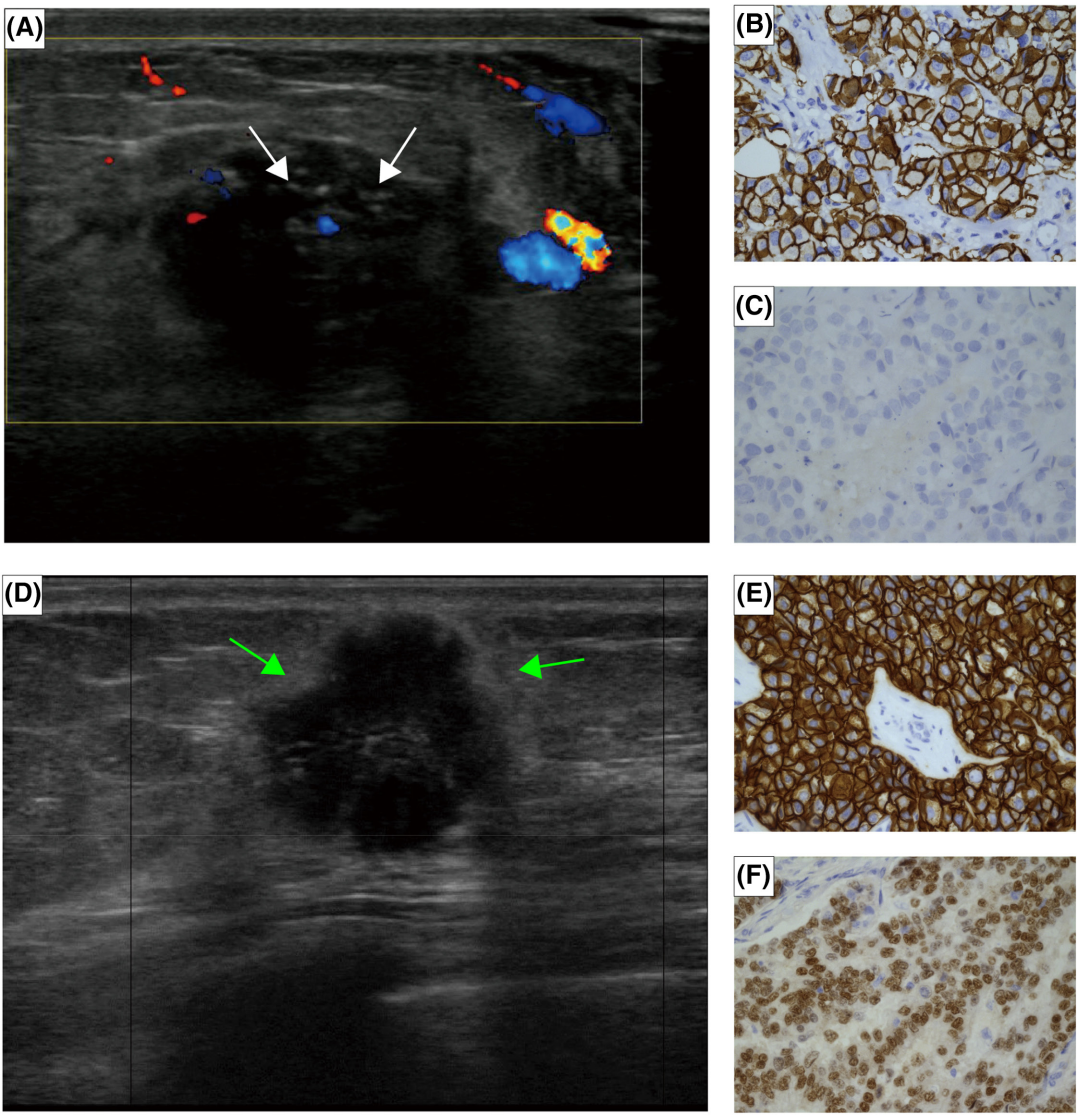
(A–C) A 59‐year‐old HR−/HER2+ breast cancer patient with axillary lymph node involvement who recurred in the 12th month after surgery. (A) The ultrasound showed calcification (white arrows) and low Adler degree. The IHC result of the patient: (B) HER2+ and (C) ER−, original magnification ×400. (D–F) A 67‐year‐old patient with HR+/HER2+ breast cancer that has not yet relapsed, with uninvolved axillary lymph node and a histological grade of 1. (D) The ultrasound showed hyperechoic halo (green arrows). The IHC result of the patient: (E) HER2+ and (F) ER+, original magnification ×400.

In HR−/HER2+ breast cancer, our results showed that informative ultrasound characteristics related to recurrence included calcification and Adler degree (Figure 7). The rapid proliferation of tumor cells depletes the blood supply, leading to tumor cell death and an increase in microenvironmental acidosis, ultimately causing the accumulation of calcium in the duct. This accumulation appears as calcification on ultrasound images.^26^ Some studies have found that breast cancer with calcification has a poor prognosis and is prone to recurrence and metastasis, which is consistent with our results in HR−/HER2+ group.^27^ However, we did not find the significant correlation between calcification and recurrence in HR+/HER2+ group, which is consistent with the results of Li et al.^28^

In addition, we found that Adler degree was negatively correlated with the recurrence of HR−/HER2+ breast cancer. Blood supply plays an irreplaceable role in the occurrence and development of tumors. Studies have confirmed that the blood supply of breast cancer is closely related to the prognosis.^29^ Chen et al. suggested that ER− breast cancer usually had rich blood flow, and showed higher Adler degree on ultrasound images.^30^ When a tumor occurs, vascular epidermal growth factor promotes the formation of new blood vessels in the tumor from the surrounding original blood vessels, leading to a poorly structured vascular network and increasing blood vessel permeability.^31^ This may provide a convenient way for drug entry and exit, thereby improving drug efficacy and reducing the probability of recurrence. Taken together, further investigation of this finding is warranted.

In HR+/HER2+ breast cancer, we found that tumors with echogenic halo had a lower risk of recurrence (Figure 7). Some studies have shown that the echogenic halo may be formed by a fibroproliferative reaction, caused by the excessive sound reflection or attenuation of the tumor relative to the surrounding tissues.^32^ This is consistent with the histopathological characteristics of tumor cells infiltrating adipose tissue, fat cells, and elastic fibers.^33^, ^34^ Thus, ultrasound images showing a hyperechoic halo suggest that fibrous tissue proliferation around the tumor inhibits cancer cell infiltration into the periphery and prevents metastasis to the axillary lymph nodes. The specific mechanism surrounding this observation requires more research and exploration.

Currently, numerous studies are assessing breast cancer prognosis using imaging features such as mammography, MRI, and ultrasound. Studies have shown that extremely low density in mammography is an independent prognostic factor.^35^ Additionally, MRI‐detected peritumoral edema has been demonstrated to be associated with worse distant metastasis‐free survival.^36^ Ultrasound plays an important role in the diagnosis and treatment of breast cancer, offering advantages such as real‐time, non‐invasive, and repeatability. Relevant studies have found that tumors with posterior acoustic shadows are associated with poor prognosis in TNBC.^37^ However, our study specifically analyzed HER2+ breast cancer based on different HR status, and informative ultrasound features were extracted for more individualized and precise prediction.

Our study innovatively established two nomograms to predict RFS in HR−/HER2+ and HR+/HER2+ patients, respectively. HER2+ breast cancer, as a heterogeneous subtype, has various clinical outcomes with different HR status.^4^, ^5^ Therefore, it is necessary to explore HER2+ breast cancer separately according to the HR status. Moreover, the two nomograms were constructed based on clinicopathological and ultrasound characteristics, which could predict the prognosis non‐invasively and quickly. Additionally, they both demonstrated a high C‐index and excellent calibration curve in both internal and external validation. Therefore, individualized prediction of RFS by nomograms would allow for improving clinical treatment plans, thereby enhancing long‐term efficacy.

Still, there are limitations to this study. First, the retrospective study design may inevitably bias the selection of patients. Second, patients were included from only two institutions and the sample size was relatively small. In future studies, it is necessary to include more patients from different institutions and diverse regions to improve the model and make it more applicable. In short, our model requires further validation through prospective studies with a larger sample size and longer follow‐up time.

## 5 CONCLUSION

In conclusion, our study has resulted in the development and verification of nomograms based on ultrasound and clinicopathological characteristics to evaluate recurrence of HER2+ breast cancer with different HR status. The combination of ultrasound and clinicopathological characteristics improves the accuracy of individualized recurrence prediction in HER2+ breast cancer patients. In the foreseeable future, it is likely that the combination of multiple disciplines will lead to the improved accuracy of individualized recurrence prediction tools.

## AUTHOR CONTRIBUTIONS

**Xudong Zhang:** Conceptualization (equal); formal analysis (equal); writing – review and editing (equal). **Hanqing Kong:** Conceptualization (equal); formal analysis (equal); writing – original draft (equal). **Xiaoxue Liu:** Data curation (equal). **Qingxiang Li:** Data curation (equal). **Xinran Fang:** Methodology (equal). **Junjia Wang:** Methodology (equal). **Zihao Qin:** Methodology (equal). **Nana Hu:** Data curation (equal). **Jiawei Tian:** Resources (equal). **Hao Cui:** Conceptualization (equal); resources (equal). **Lei Zhang:** Conceptualization (equal); resources (equal).

## FUNDING INFORMATION

This study was funded by the National Natural Science Foundation of China (grant number 82272001, 82102072, and 82371984), Outstanding Youth Program of Heilongjiang Natural Science Foundation (YQ2022148).

## CONFLICT OF INTEREST STATEMENT

The authors declare that they have no competing interests.

## ETHICS STATEMENT

This study was approved by the institutional ethics committee of Harbin medical university (approval number, KY‐2016‐127).

## CONSENT FOR PUBLICATION

Informed consent was not required because this study was a retrospective report of cases, which is a retrospective analysis of clinical data. The need of informed consent was waived by the ethical committee of the Harbin medical university.

## Supporting information


Data S1.

## Data Availability

The imaging data and code that support the findings of this study are available from the corresponding author upon request.
