# Supplementary material for: Nomograms for predicting recurrence of HER2‐positive breast cancer with different HR status based on ultrasound and clinicopathological characteristics
Source: Cancer Med. 2024 Sep 9;13(17):e70146. doi: 10.1002/cam4.70146 (PMC11381954; doi:10.1002/cam4.70146)
Supplement: Supplementary file 1 — Data S1. [file CAM4-13-e70146-s001.docx]

| **Table S1.** Ultrasound characteristics of the breast lesions. | |
| --- | --- |
| Characteristic | Definition |
| **Shape** |  |
| Regular | Oval, spherical or round |
| Irregular | Not round or oval |
| **Orientation** |  |
| Parallel | Long axis of lesion parallels the skin line |
| Vertical | Long axis, not oriented along the skin line |
| **Boundary** |  |
| Circumscribed | Sharp demarcation between tumor and surrounding tissue |
| Indistinct | Not circumscribed, blurry, exact position of the margin is hardly to define |
| **Margin** |  |
| Smooth | Smooth, even margin without any irregular |
| Lobulate | Short cycle undulations impart a scalloped appearance to the margin of the mass |
| **Posterior acoustic pattern** |  |
| No enhancement | No change or shadowing |
| Enhancement | Increased posterior echo |
| **Calcification** |  |
| Absent | No punctuated extensively hyperechoic foci |
| Present | Punctuated extensively hyperechoic foci |
| **Echogenic halo** |  |
| Absent | No thin capsule or echoic halo |
| Present | Blurred, irregular hyperechoic rim around the lesion |
| **Internal echo** |  |
| Hypoechoic | Hypoechoic compared to surrounding tissue |
| Mixed-echoic | Heterogeneous or mixed echo |

**Supplementary** **material**

| **Table S2.** The training cohort and the validation cohort of HR-/HER2+ | | | |
| --- | --- | --- | --- |
| Characteristic | Training cohort  (n=178) | Validation cohort  (n=46) | *P* Value |
| **Age** |  |  | 0.206 |
| ≤35 | 124 (69.66) | 37 (80.43) |  |
| >35 | 54 (30.34) | 9 (19.57) |  |
| **Tumor size** |  |  | 0.606 |
| ≤2cm | 99 (55.62) | 23 (50) |  |
| >2cm | 79 (44.38) | 23 (50) |  |
| **Histological grade** |  |  | 0.822 |
| 1-2 | 87 (48.88) | 21 (45.65) |  |
| 3 | 91 (51.12) | 25 (54.35) |  |
| **Axillary nodal status** |  |  | 0.784 |
| No involvement | 107 (60.11) | 26 (56.52) |  |
| Involvement | 71 (39.89) | 20 (43.48) |  |
| **Surgery type** |  |  | 1.000 |
| BCT | 5 (2.81) | 1 (2.17) |  |
| Mast | 173 (97.19) | 45 (97.83) |  |
| **Family history of breast cancer** |  |  | 0.312 |
| Absent | 95 (53.37) | 29 (63.04) |  |
| Present | 83 (46.63) | 17 (36.96) |  |
| **Menstrual history** |  |  | 1.000 |
| Normal | 162 (91.01) | 42 (91.3) |  |
| Abnormal | 16 (8.99) | 4 (8.7) |  |
| **Lactation history** |  |  | 0.689 |
| Absent | 105 (58.99) | 25 (54.35) |  |
| Present | 73 (41.01) | 21 (45.65) |  |
| **Ki67** |  |  | 1.000 |
| <14 | 89 (50) | 23 (50) |  |
| >14 | 89 (50) | 23 (50) |  |
| **P53** |  |  | 1.000 |
| Negative | 97 (54.49) | 25 (54.35) |  |
| Positive | 81 (45.51) | 21 (45.65) |  |
| **CK5/6** |  |  | 0.647 |
| Negative | 102 (57.3) | 24 (52.17) |  |
| Positive | 76 (42.7) | 22 (47.83) |  |
| **E-cadherin** |  |  | 0.348 |
| Negative | 104 (58.43) | 31 (67.39) |  |
| Positive | 74 (41.57) | 15 (32.61) |  |
| **CEA** |  |  | 1.000 |
| Negative | 99 (55.62) | 26 (56.52) |  |
| Positive | 79 (44.38) | 20 (43.48) |  |
| **CA153** |  |  | 0.202 |
| Negative | 75 (42.13) | 14 (30.43) |  |
| Positive | 103 (57.87) | 32 (69.57) |  |
| **Shape** |  |  | 1.000 |
| Regular | 92 (51.69) | 24 (52.17) |  |
| Irregular | 86 (48.31) | 22 (47.83) |  |
| **Orientation** |  |  | 0.882 |
| Parallel | 96 (53.93) | 26 (56.52) |  |
| Vertical | 82 (46.07) | 20 (43.48) |  |
| **Boundary** |  |  | 0.342 |
| Circumscribed | 23 (12.92) | 3 (6.52) |  |
| Indistinct | 155 (87.08) | 43 (93.48) |  |
| **Margin** |  |  | 0.573 |
| Smooth | 86 (48.31) | 25 (54.35) |  |
| Lobulate | 92 (51.69) | 21 (45.65) |  |
| **Posterior acoustic pattern** |  |  | 0.847 |
| No enhancement | 125 (70.22) | 31 (67.39) |  |
| Enhancement | 53 (29.78) | 15 (32.61) |  |
| **Calcification** |  |  | 0.913 |
| Absent | 116 (65.17) | 31 (67.39) |  |
| Present | 62 (34.83) | 15 (32.61) |  |
| **Echogenic halo** |  |  | 0.739 |
| Absent | 21 (11.8) | 4 (8.7) |  |
| Present | 157 (88.2) | 42 (91.3) |  |
| **Internal echo** |  |  | 0.180 |
| Hypoechoic | 80 (44.94) | 15 (32.61) |  |
| Mixed-echoic | 98 (55.06) | 31 (67.39) |  |
| **Adler degree** |  |  | 0.312 |
| 0-1 | 67 (37.64) | 13 (28.26) |  |
| 2-3 | 111 (62.36) | 33 (71.74) |  |
| **BI-RADS** |  |  | 0.025 |
| ≤3 | 160 (89.89) | 35 (76.09) |  |
| >3 | 18 (10.11) | 11 (23.91) |  |

| **Table S3.** The training cohort and the validation cohort of HR+/HER2+ | | | |
| --- | --- | --- | --- |
| Characteristic | Training cohort  (n=277) | Validation cohort  (n=69) | *P* Value |
| **Age** |  |  | 0.553 |
| ≤35 | 139 (50.18) | 38 (55.07) |  |
| >35 | 138 (49.82) | 31 (44.93) |  |
| **Tumor size** |  |  | 0.372 |
| ≤2cm | 183 (66.06) | 41 (59.42) |  |
| >2cm | 94 (33.94) | 28 (40.58) |  |
| **Histological grade** |  |  | 0.750 |
| 1-2 | 224 (80.87) | 54 (78.26) |  |
| 3 | 53 (19.13) | 15 (21.74) |  |
| **Axillary nodal status** |  |  | 0.527 |
| No involvement | 98 (35.38) | 21 (30.43) |  |
| Involvement | 179 (64.62) | 48 (69.57) |  |
| **Surgery type** |  |  | 0.625 |
| BCT | 19 (6.86) | 3 (4.35) |  |
| Mast | 258 (93.14) | 66 (95.65) |  |
| **Family history of breast cancer** |  |  | 0.669 |
| Absent | 214 (77.26) | 51 (73.91) |  |
| Present | 63 (22.74) | 18 (26.09) |  |
| **Menstrual history** |  |  | 0.449 |
| Normal | 161 (58.12) | 36 (52.17) |  |
| Abnormal | 116 (41.88) | 33 (47.83) |  |
| **Lactation history** |  |  | 0.327 |
| Absent | 81 (29.24) | 25 (36.23) |  |
| Present | 196 (70.76) | 44 (63.77) |  |
| **Ki67** |  |  | 0.994 |
| <14 | 99 (35.74) | 24 (34.78) |  |
| >14 | 178 (64.26) | 45 (65.22) |  |
| **P53** |  |  | 0.796 |
| Negative | 75 (27.08) | 17 (24.64) |  |
| Positive | 202 (72.92) | 52 (75.36) |  |
| **CK5/6** |  |  | 0.554 |
| Negative | 150 (54.15) | 34 (49.28) |  |
| Positive | 127 (45.85) | 35 (50.72) |  |
| **E-cadherin** |  |  | 1.000 |
| Negative | 168 (60.65) | 42 (60.87) |  |
| Positive | 109 (39.35) | 27 (39.13) |  |
| **CEA** |  |  | 0.831 |
| Negative | 151 (54.51) | 36 (52.17) |  |
| Positive | 126 (45.49) | 33 (47.83) |  |
| **CA153** |  |  | 0.282 |
| Negative | 143 (51.62) | 30 (43.48) |  |
| Positive | 134 (48.38) | 39 (56.52) |  |
| **Shape** |  |  | 1.000 |
| Regular | 74 (26.71) | 19 (27.54) |  |
| Irregular | 203 (73.29) | 50 (72.46) |  |
| **Orientation** |  |  | 0.627 |
| Parallel | 141 (50.9) | 38 (55.07) |  |
| Vertical | 136 (49.1) | 31 (44.93) |  |
| **Boundary** |  |  | 0.247 |
| Circumscribed | 56 (20.22) | 19 (27.54) |  |
| Indistinct | 221 (79.78) | 50 (72.46) |  |
| **Margin** |  |  | 0.315 |
| Smooth | 168 (60.65) | 47 (68.12) |  |
| Lobulate | 109 (39.35) | 22 (31.88) |  |
| **Posterior acoustic pattern** |  |  | 0.053 |
| No enhancement | 84 (30.32) | 30 (43.48) |  |
| Enhancement | 193 (69.68) | 39 (56.52) |  |
| **Calcification** |  |  | 0.029 |
| Absent | 73 (26.35) | 28 (40.58) |  |
| Present | 204 (73.65) | 41 (59.42) |  |
| **Echogenic halo** |  |  | 0.991 |
| Absent | 83 (29.96) | 20 (28.99) |  |
| Present | 194 (70.04) | 49 (71.01) |  |
| **Internal echo** |  |  | 0.306 |
| Hypoechoic | 142 (51.26) | 30 (43.48) |  |
| Mixed-echoic | 135 (48.74) | 39 (56.52) |  |
| **Adler degree** |  |  | 0.674 |
| 0-1 | 210 (75.81) | 50 (72.46) |  |
| 2-3 | 67 (24.19) | 19 (27.54) |  |
| **BI-RADS** |  |  | 0.176 |
| ≤3 | 145 (52.35) | 43 (62.32) |  |
| >3 | 132 (47.65) | 26 (37.68) |  |

| **Table S4.**  Univariate and multivariate analysis of clinicopathological and ultrasound characteristics for prognostic prediction in HR-/HER2+. | | | | |
| --- | --- | --- | --- | --- |
| Characteristic | Univariate regression | | Multivariate regression | |
|  | HR (95% CI) | *P*Value | HR (95% CI) | *P*Value |
| Age | 1.169(0.587-2.327) | 0.657 |  |  |
| Menstrual history | 1.432(0.558-3.676) | 0.456 |  |  |
| Family history | 1.309(0.687-2.494) | 0.414 |  |  |
| Lactation history | 2.152(1.122-4.124) | 0.021 | 2.444 (0.570-10.471) | 0.229 |
| Surgery type | 0.841(0.115-6.145) | 0.864 |  |  |
| Histological grade | 1.298(0.677-2.487) | 0.432 |  |  |
| Axillary nodal status | 2.705(1.402-5.221) | 0.003 | 2.447 (1.226-4.884) | 0.011 |
| Ki67 | 0.968(0.508-1.845) | 0.921 |  |  |
| P53 | 0.945(0.493-1.811) | 0.865 |  |  |
| CK5/6 | 1.031(0.538-1.975) | 0.928 |  |  |
| E-cadherin | 0.85(0.438-1.653) | 0.633 |  |  |
| CEA | 1.853(0.967-3.553) | 0.063 | 1.498 (0.763-2.940) | 0.240 |
| CA153 | 0.945(0.493-1.812) | 0.866 |  |  |
| Tumor Size | 1.782(0.930-3.416) | 0.082 | 0.753 (0.177-3.202) | 0.701 |
| Shape | 0.968(0.508-1.845) | 0.922 |  |  |
| Orientation | 1.029(0.539-1.964) | 0.931 |  |  |
| Boundary | 1.170(0.415-3.304) | 0.766 |  |  |
| Margin | 0.972(0.510-1.853) | 0.932 |  |  |
| Posterior acoustic pattern | 1.299(0.661-2.552) | 0.448 |  |  |
| Calcification | 2.079(1.091-3.963) | 0.026 | 2.342 (1.206-4.551) | 0.012 |
| Echogenic halo | 1.361(0.418-4.437) | 0.609 |  |  |
| Internal echo | 1.243(0.645-2.396) | 0.516 |  |  |
| Adler degree | 0.274(0.140-0.539) | <0.001 | 0.390 (0.190-0.798) | 0.010 |
| BI-RADS | 1.671(0.697-4.005) | 0.250 |  |  |

| **Table S5.**  Univariate and multivariate analysis of clinicopathological and ultrasound characteristics for prognostic prediction in HR+/HER2+. | | | | |
| --- | --- | --- | --- | --- |
| Characteristic | Univariate regression | | Multivariate regression | |
|  | HR (95% CI) | *P*Value | HR (95% CI) | *P*Value |
| Age | 0.553(0.286-1.069) | 0.078 | 0.703(0.338-1.463) | 0.346 |
| Menstrual history | 1.130(0.596-2.142) | 0.708 |  |  |
| Family history | 1.133(0.536-2.393) | 0.744 |  |  |
| Lactation history | 0.898(0.453-1.780) | 0.758 |  |  |
| Surgery type | 0-Inf | 0.996 |  |  |
| Histological grade | 2.565(1.327-4.959) | 0.005 | 3.235(1.635-6.403) | <0.001 |
| Axillary nodal status | 5.357(1.900-15.102) | 0.002 | 6.367(2.236-18.133) | <0.001 |
| Ki67 | 1.245(0.628-2.468) | 0.529 |  |  |
| P53 | 1.030(0.500-2.120) | 0.936 |  |  |
| CK5/6 | 1.251(0.662-2.363) | 0.490 |  |  |
| E-cadherin | 1.133(0.595-2.157) | 0.704 |  |  |
| CEA | 1.378(0.729-2.605) | 0.324 |  |  |
| CA153 | 1.228(0.649-2.321) | 0.528 |  |  |
| Tumor Size | 1.198(0.620-2.316) | 0.591 |  |  |
| Shape | 0.952(0.462-1.960) | 0.894 |  |  |
| Orientation | 1.217(0.644-2.301) | 0.545 |  |  |
| Boundary | 1.397(0.584-3.341) | 0.453 |  |  |
| Margin | 1.227(0.647-2.326) | 0.531 |  |  |
| Posterior acoustic pattern | 0.948(0.479-1.880) | 0.879 |  |  |
| Calcification | 1.187(0.562-2.508) | 0.653 |  |  |
| Echogenic halo | 0.358(0.190-0.677) | 0.002 | 0.417(0.212-0.820) | 0.011 |
| Internal echo | 2.851(1.414-5.747) | 0.003 | 2.244(1.065-4.728) | 0.034 |
| Adler degree | 1.734(0.887-3.391) | 0.108 | 1.763(0.848-3.667) | 0.129 |
| BI-RADS | 0.788(0.414-1.500) | 0.467 |  |  |
